# Supplementary material for: Patterns of Intron Gain and Loss in Fungi
Source: PLoS Biol. 2004 Nov 30;2(12):e422. doi: 10.1371/journal.pbio.0020422 (PMC532390; doi:10.1371/journal.pbio.0020422)
Supplement: Table S1 — Also available at http://genes.mit.edu/NielsenEtAl/. (4.3 MB ZIP). [file pbio.0020422.st001.zip › NielsenEtAl/html/162.html]

AN8046.1.NCU03639.1.MG07016.1.FG05906.1


```
 CLUSTAL W (1.82) Multiple Sequence Alignments - Introns Inserted


Sequence 1: MG07016.1	348 aa
Sequence 2: FG05906.1	349 aa
Sequence 3: NCU03639.1	337 aa
Sequence 4: AN8046.1	294 aa
Alignment Length: 360 aa
Number Identitical Residues: 65 aa
Alignment Score (without introns) 4866


MG07016.1 	MRFPSVLTLLATALTCSASVLPAGLTYTKTVEGRD~V~T~VSETDLDNFRFYAQYSAATY
NCU03639.1	MKASLFLASALAATGLAAP------STSPNMAKRQ~F1V~VTSSELSKLSYWAEQAATSY
FG05906.1 	MRLLSLLSVVTLAVASPLSVE----EYAKALDERA1V~S~VSTTDFGNFKFYIQHGAAAY
AN8046.1  	MIRLGYSAIFVALAGLAVAAP-------APLNRR-~-~D1VSTEALNQLTLFAEYSAASY
          	*      :        . .           :  *      *:   :.::  : : .*::*

MG07016.1 	CNDAA-ASGAAVACSNDGCPAVVANGAKIIRSLN~Q~D-TSTNTAGYLALDPKRKNIVLA
NCU03639.1	CNSNN-AAGDLIACSNNVCPTLVSNKAVTIASFA2S~G-TATDIHGLVSVDPVKKVITVS
FG05906.1 	CNSEA-PAGAKVTCSGNGCPTVQSNGATIVASFT2G~--SKTGIGGYVATDPTRKEIVVS
AN8046.1  	CTPNIGSVGDKLTCASGNCPTVEAADTTTLAEFY~Q2ENEYGDVAGFLAADTTNELLVLS
          	*.   .. *  ::*:.. **:: :  :  : .:     .   .  * :: *. .: :.::

MG07016.1 	LRGSTSLRNWITN~LTFLWTRCD-FVQDCKLHTGFATAWSQVQADVLAAIADAKAQNPDY
NCU03639.1	FRGSSSVRNWITD2VVFVKSSCDELVSGCLIHTGFYTAWREVATKVTAAVQSAKAAYPSY
FG05906.1 	FRGSINIRNWLTN~LDFDQDDCS-LTSGCGVHSGFQNAWNEISAAATAAVAKARKANPSF
AN8046.1  	FRGSRTIDTWIAN~LDFGLESVEEICSGCKAHGGFWKAWQVVADSLTSAIESATATYPGY
          	:*** .: .*::: : *     ..: ..*  * ** .**  :     :*: .*    *.:

MG07016.1 	TVVVTGHSLGGAVATVAGVYLRQLGYPVEV~YTYGSPRIGNQEFVQWVSTQAGNVEYRVT
NCU03639.1	SIGVTGHSLGGAVATVAAAYLRKAGYTADL~YTFGSPRVGNEAFAAFTTEQSG-DEYRVT
FG05906.1 	KVVSVGHSLGGAVATLAGANLRVGGTPLDI~YTYGSPRVGNTQLAAFVSNQAG-GEFRVT
AN8046.1  	AIVFTGHSFGGALATLGAAQLRKAGYAIEL0YPYGSPRVGNEALAQYITDQGA--NYRVT
          	 :  .***:***:**:... **  * . :: *.:****:**  :. : : *..  ::***

MG07016.1 	HIDDPVPRLPPIFLGYRHVTPEYWLNSGTSNTVNYTVADIKVCEGFANINCNGGSLGLDT
NCU03639.1	HENDPVPRLPPISFNYRHTSPEWWIQAAVP-----TTSQVKICPGYASIDCNAATLGFKR
FG05906.1 	NAKDPVPRLPPLIFGYRHTSPEYWLSGSGGDKIDYTINDVKVCEGAANLRCNGGTLGLDI
AN8046.1  	HTNDIVPRLPPMLLGFSHLSPEYWITSDNE--VTPTTTDIQVIEGVGSRDGNAGEAAQSV
          	: .* ******: :.: * :**:*: .        *  ::::  * ..   *..  . . 

MG07016.1 	NAHLYYLTDMIACGSNKFVFRR---------DDANAISDAELEQRLTMYAQMDREFVAAL
NCU03639.1	DDHLHYFEDIAGCSPGGFNWKR-----AESNVASSDVSDEELESRLNQWVVEDVNFVNSN
FG05906.1 	DAHLHYFQATDACSAGGISWRRYRSAKRESISERATMTDAELEKKLNSYVEMDKEYIKT-
AN8046.1  	EAHSWYLIDITACQ----------------------------------------------
          	: *  *:    .*                                               

MG07016.1 	EANKTVA
NCU03639.1	DLH----
FG05906.1 	HANRSS-
AN8046.1  	-------
          	
```
